# GIMDA: Graphlet interaction‐based MiRNA‐disease association prediction

**DOI:** 10.1111/jcmm.13429

**Published:** 2017-12-22

**Authors:** Xing Chen, Na‐Na Guan, Jian‐Qiang Li, Gui‐Ying Yan

**Affiliations:** ^1^ School of Information and Control Engineering China University of Mining and Technology Xuzhou China; ^2^ College of Computer Science and Software Engineering Shenzhen University Shenzhen China; ^3^ Academy of Mathematics and Systems Science Chinese Academy of Sciences Beijing China

**Keywords:** miRNA, disease, miRNA‐disease association, graphlet interaction

## Abstract

MicroRNAs (miRNAs) have been confirmed to be closely related to various human complex diseases by many experimental studies. It is necessary and valuable to develop powerful and effective computational models to predict potential associations between miRNAs and diseases. In this work, we presented a prediction model of Graphlet Interaction for MiRNA‐Disease Association prediction (GIMDA) by integrating the disease semantic similarity, miRNA functional similarity, Gaussian interaction profile kernel similarity and the experimentally confirmed miRNA‐disease associations. The related score of a miRNA to a disease was calculated by measuring the graphlet interactions between two miRNAs or two diseases. The novelty of GIMDA lies in that we used graphlet interaction to analyse the complex relationships between two nodes in a graph. The AUCs of GIMDA in global and local leave‐one‐out cross‐validation (LOOCV) turned out to be 0.9006 and 0.8455, respectively. The average result of five‐fold cross‐validation reached to 0.8927 ± 0.0012. In case study for colon neoplasms, kidney neoplasms and prostate neoplasms based on the database of HMDD V2.0, 45, 45, 41 of the top 50 potential miRNAs predicted by GIMDA were validated by dbDEMC and miR2Disease. Additionally, in the case study of new diseases without any known associated miRNAs and the case study of predicting potential miRNA‐disease associations using HMDD V1.0, there were also high percentages of top 50 miRNAs verified by the experimental literatures.

## Introduction

MicroRNAs (miRNAs) are a category of single‐stranded non‐coding RNAs which contain about 20~25 nucleotides in length. They play an important role in the regulation of gene expression at the post‐transcriptional and translational level by binding to the 3′untranslated regions (UTRs) of the target mRNAs 1, 2, 3, 4. MiRNAs have been detected in various organisms ranging from viruses and microbes to eukaryotic organisms and their number have reached to 28645 (2588 for human) in the latest release of miRBase 5, 6, 7. Many studies have implied that miRNAs participate in manifold biological processes, such as cell proliferation 8, development 9, apoptosis 10, differentiation 11, signal transduction 12 and so on. Therefore, more and more evidences have confirmed that miRNAs are closely related to many kinds of human diseases 13, 14, 15, 16. For example, Heegaard *et al*. 17 developed a method to use quantitative real‐time PCR (qRT‐PCR) to measure the circulating levels of 30 miRNAs and found that the expressions of miR‐146b, miR‐221, let‐7a, miR‐155, miR‐17‐5p, miR‐27a and miR‐106a were significantly reduced in the serum of non‐small cell lung cancer (NSCLC) cases although miR‐29c was much increased. Meanwhile, they also obtained evidence that expression of let‐7b was associated with prognosis in NSCLC. Besides, authors of Ref. 18 and 19 reported a connection between miR‐137, miR‐181c, miR‐9, miR‐29a/b and Alzheimer's disease (AD) and concluded that these miRNAs could be treated as diagnostic markers for AD. In addition, miR‐17~92 cluster was found to be up‐regulated in polycystic kidney disease (PKD) and could be identified as a therapeutic target in PKD 20. Most recently, using qRT‐PCR analyses, studies have shown that peripheral blood miRNA‐720 and miRNA‐1246 might be considered as a promoting factor in the development of multiple myeloma (MM) and hence could be used as diagnostic factor, therapeutic effect evaluator and prognostic indicator in the prognosis of MM 21. Although experiments have achieved many significant results, they are expensive and time‐consuming. Therefore, it is urgent to develop computational models to guide the biological experiments by inferring latent miRNA‐disease associations based on the large numbers of miRNA‐associated data sets 22, 23, 24, 25, 26, 27, 28, 29, 30, 31, 32, 33, 34, 35, 36, 37, 38.

In fact, there have been plenty of computational models developed in the past few years to predict potential miRNA‐disease associations 39, 40, 41. Based on the notion that functionally related miRNAs tend to be associated with phenotypically similar diseases, Jiang *et al*. 42 proposed a hypergeometric distribution‐based computational model. However, this model only uses the information of the direct network neighbours of miRNAs, ignoring those indirectly linked miRNAs. Besides, this model uses miRNA‐target interactions which have a high rate of false‐positive and false‐negative samples. Moreover, Xu *et al*. 43 constructed a miRNA target‐dysregulated network and introduced a miRNA prioritization method that did not depend on the known miRNA‐disease associations but the similarity between the miRNA targets and disease genes. In addition, Shi *et al*. 44 presented a computational framework to identify miRNA‐disease associations using random walk on protein–protein interaction (PPI) network. Through integrating miRNA‐target interaction, disease–gene associations and PPIs, they found out the co‐regulated modules of miRNA and disease and thus the connection between them. Mørk *et al*. 45 further proposed a miRPD model that combined known and predicted miRNA–protein associations with protein–disease associations to infer miRNA–Protein‐Disease associations. The aforementioned models relied much on miRNA‐target interactions, which usually had high false‐positive and false‐negative ratios; hence, they did not perform very well.

Furthermore, Xuan *et al*. 46 developed a method of Human Disease‐MiRNA associations Prediction (HDMP), by considering the weighted *k* most similar neighbours of miRNAs, where the members in the same miRNA family or cluster were assigned higher weight. However, this model cannot be applied to new diseases without any known related miRNAs as it needs neighbours of miRNAs and its prediction accuracy is limited because of dependence on the algorithm adopting local similarity measure. Also, Xuan *et al*. 47 devised another computational model based on random walk on miRNA functional similarity network. They exploited the miRNA similarity, the disease similarity, the known miRNA‐disease associations, the topology information of the bilayer network, as well as the information from different layers of network to predict disease miRNA candidates. In particular, this method is adoptable to predict potential miRNAs for diseases without known related miRNAs. Chen *et al*. 48, for the first time, presented a global network similarity measure‐based model of Random Walk with Restart for MiRNA‐Disease Association (RWRMDA). This model searches for potential miRNA‐disease associations through applying random walk to the miRNA–miRNA functional similarity network. However, it fails to predict related miRNAs of new diseases that have no known association. To overcome this limitation, Chen *et al*. 25 developed another model of Regularized Least Squares for MiRNA‐Disease Association prediction (RLSMDA). It is a semi‐supervised and global method based on regularized least squares, which does not need negative samples. Moreover, a model of restricted Boltzmann machine for multiple types of miRNA‐disease association prediction was proposed by Chen *et al*. 22. This is the first model that can infer the association types between miRNAs and diseases, which includes evidences of miRNA‐target interactions, circulation, epigenetics and genetics. However, it has many complex parameters to train. Another model named Within and Between Score for MiRNA‐Disease Association prediction (WBSMDA) was presented by Chen *et al*. 24, in which Gaussian interaction profile kernel similarity of miRNAs and diseases were calculated and integrated with miRNA functional similarity or disease semantic similarity. It is mentionable that WBSMDA is valid not only for new diseases with no known related miRNAs but also for new miRNAs without known related diseases. Moreover, a method of Heterogeneous Graph Inference for MiRNA‐Disease Association prediction (HGIMDA) was developed through combining miRNA functional similarity, disease semantic similarity, Gaussian interaction profile kernel similarity and known miRNA‐disease associations to construct a heterogeneous graph 49. It computed the potential association probabilities between miRNAs and diseases by summarizing all paths with the length equal to three within the heterogeneous graph. Recently, Li *et al*. 50 used matrix completion algorithm and single value thresholding (SVT) to establish a computational model of Matrix Completion for MiRNA‐Disease Association prediction (MCMDA) based on the known miRNA‐disease associations. This model can update the low‐rank miRNA‐disease interaction matrix quickly, and it does not need the negative samples. Besides, Chen *et al*. 51 developed another model of Ranking‐based KNN for MiRNA‐Disease Association Prediction (RKNNMDA), which uses KNN algorithm to search for the *k*‐nearest neighbours of miRNAs and diseases according to the similarity scores. Using the SVM Ranking model, they ranked the *k*‐nearest neighbours and obtained the most possible miRNA‐disease associations. This method can also be applied to new diseases without any known related miRNAs.

As mentioned above, it is very time‐consuming and expensive to search for new disease‐related miRNAs through biological experiments, and existing computational models cannot absolutely satisfy the demand for prediction. Therefore, in this study, we developed a model of Graphlet Interaction for MiRNA‐Disease Association prediction (GIMDA), where all miRNAs and diseases are represented as nodes of the graph, respectively. We considered 28 types of isomers for each graphlet interaction between two nodes. By counting the numbers of graphlet interaction isomers, the related score of a miRNA to a disease can be computed in miRNA graph and in disease graph, respectively. We implemented leave‐one‐out cross‐validation (LOOCV) and fivefold cross‐validation to estimate the performance of GIMDA. The AUCs of global and local LOOCV are 0.9006 and 0.8455, respectively. And the average AUC of fivefold cross‐validation reaches to 0.8927 ± 0.0012. In addition, twofold, threefold and fourfold cross‐validations were implemented and obtained good results which have indicated the robustness of the model. In case study for colon neoplasms, kidney neoplasms and prostate neoplasms, the top 50 miRNAs predicted by GIMDA using HMDD V2.0 52 as known associations were validated based on dbDEMC 53 and miR2Disease 54. The confirmed result numbers of these three diseases are 45, 45, 41, respectively. In the case study for new diseases without any known associated miRNAs, 50 of top 50 miRNAs that were predicted to be related to hepatocellular carcinoma are validated by HMDD V2.0, dbDEMC or miR2Disease. Furthermore, using HMDD V1.0, the case study of oesophageal neoplasms also shows a high validation percentage. All above experimental results suggest that GIMDA is a reliable model and can be used to predict potential associations between miRNAs and diseases.

## Materials and methods

### Human miRNA‐disease associations

In this manuscript, the known miRNA‐disease associations were downloaded from HMDD V2.0 52. The total number of associations is 5430, referring to 495 miRNAs and 383 diseases. Based on the known data, an adjacency matrix *A* was constructed to represent the relations between all miRNAs and all diseases. The element *A*(*i*,* j*) was set to be 1 if there was an association between disease *d*(*i*) and miRNA *m*(*j*), 0 otherwise. The variables *m* and *n* denote the total numbers of miRNAs and diseases in the association data set, respectively.

### MiRNA functional similarity

The miRNA functional similarity was calculated using the method proposed by Wang *et al*. 39 and could be obtained by downloading from the website: http://www.cuilab.cn/files/images/cuilab/misim.zip. The similarity data set was transformed into a square matrix *FS* in which the element *FS*(*i*,* j*) denoted the similarity value between miRNA *m*(*i*) and *m*(*j*).

### Disease semantic similarity model 1

According to several computing models 24, 33, 35, 36, we used a Directed Acyclic Graph (DAG) to describe a disease. Here, a disease *D* can be represented by *DAG*(*D*) = (*D*,* T*(*D*), *E*(*D*)), where *T*(*D*) is the node set consisting of the disease *D* and its ancestor nodes, and *E*(*D*) is the edge set including the direct edges from parent nodes to child nodes. And then, the semantic value of the disease *D* is given by:

(1) $$DV1\left(D\right)=\sum_{d\in T\left(D\right)}D1_{D}\left(d\right)$$

where $D1_{D}\left(d\right)$ denotes the contribution from node d, which can be calculated in following way:

(2) $$\left\{\begin{array}{cc} D1_{D}\left(d\right)=1 & ifd=D\\ D1_{D}\left(d\right)=\text{max}\left\{\Delta *D1_{D}\left(d^{\prime }\right)|d^{\prime }\in \text{children of}\;d\right\} & ifd\neq D \end{array}\right.$$

where Δ is the semantic contribution decay factor. The contribution from disease *D* to its own semantic value is 1, and the contribution of other disease *d* decreases by a factor Δ as the distance between *d* and *D* increases.

Under the fact that the semantic similarity between two diseases is directly proportional to the shared part of their DAGs, the semantic similarity between disease *d*(*i*) and *d*(*j*) can be defined as follows:

(3) $$SS1\left(i,j\right)=\frac{\sum_{t\in T\left(d\left(i\right)\right)\cap T\left(d\left(j\right)\right)}\left(D1_{d\left(i\right)}\left(t\right)+D1_{d\left(j\right)}\left(t\right)\right)}{DV1\left(d\left(i\right)\right)+DV1\left(d\left(j\right)\right)}$$

### Disease semantic similarity model 2

In the first model, the diseases in the same layer of *DAG* (*D*) contribute equally to the semantic value of *D*. However, it is obvious that, for *DAG*(*D*), the disease appearing in less disease DAGs is more specific compared with other diseases in the same layer 46. In order to highlight the contributions from more specific diseases, we defined the contribution of disease *d* to the semantic value of disease *D* as follows 28

(4) $$D2_{D}\left(d\right)=-\text{log}\left[\frac{\text{the number of DAGs including}\;d}{\text{the number of diseases}}\right]$$

Then, the semantic value of disease *D* can be defined in the similar way as model 1:

(5) $$DV2\left(D\right)=\sum_{d\in T\left(D\right)}D2_{D}\left(d\right)$$

Therefore, the semantic similarity between disease *d*(*i*) and *d*(*j*) can be calculated by:

(6) $$SS2\left(i,j\right)=\frac{\sum_{t\in T\left(d\left(i\right)\right)\cap T\left(d\left(j\right)\right)}\left(D2_{d\left(i\right)}\left(t\right)+D2_{d\left(j\right)}\left(t\right)\right)}{DV2\left(d\left(i\right)\right)+DV2\left(d\left(j\right)\right)}$$

### Gaussian interaction profile kernel similarity for diseases

Based on the notion that functionally similar miRNAs are usually associated with similar diseases, we can use the known associations between miRNAs and diseases to construct the Gaussian interaction profile kernel similarity for diseases. Firstly, to describe the interaction profile of disease *d*(*i*), a binary vector *IP*(*d*(*i*)) is defined by observing whether each miRNA is related to *d*(*i*) or not, that is, *IP*(*d*(*i*)) is the *i*th row of the adjacency matrix *A*. In this case, the Gaussian interaction profile kernel similarity between disease *d*(*i*) and *d*(*j*) can be given by:

(7) $$KD\left(i,j\right)=\text{exp}\left(-\gamma_{d}\left|\right|IP\left(d\left(i\right)\right)-IP\left(d\left(j\right)\right){\left|\right|}^{2}\right)$$

where, γ_*d*_ is a parameter used to control the kernel bandwidth, which can be computed by normalizing the original bandwidth *γ*_*d*_′ as follows:

(8) $$\gamma_{d}=\frac{\gamma_{d}^{\prime }}{\left(\frac{1}{n}\sum_{i=1}^{n}||IP(d\left(i\right){\left|\right|}^{2}\right)}$$

### Gaussian interaction profile kernel similarity for miRNAs

The Gaussian interaction profile of the miRNA is defined similarly to the disease. Accordingly, the Gaussian interaction profile kernel similarity between miRNA *m*(*i*) and *m*(*j*) can be calculated as:

(9) $$KM\left(i,j\right)=\text{exp}\left(-\gamma_{m}\left|\right|IP\left(m\left(i\right)\right)-IP\left(m\left(j\right)\right){\left|\right|}^{2}\right)$$

where

(10) $$\gamma_{m}=\frac{\gamma_{m}^{\prime }}{\left(\frac{1}{m}\sum_{i=1}^{m}||IP(m\left(i\right){\left|\right|}^{2}\right)}$$

### Integrated similarity for miRNAs and diseases

The integrated similarity of diseases can be obtained by combining the Gaussian interaction profile kernel similarity with the semantic similarity which is the average of *SS*1 and *SS*2. The combination is performed in the following way:

(11) $$SD\left(i,j\right)=\left\{\begin{array}{cc} \frac{SS1(i,j)+SS2(i,j)}{2} & d(i)andd(j)hassemanticsimilarity\\ KD(i,j) & \mathrm{otherwise} \end{array}\right.$$

Similarly, the integrated similarity of miRNAs is combined as follows:

(12) $$SM\left(i,j\right)=\left\{\begin{array}{cc} FS(i,j) & m(i)andm(j)hasfunctionalsimilarity\\ KM(i,j) & \mathrm{otherwise} \end{array}\right.$$

### GIMDA

**Figure 1:**
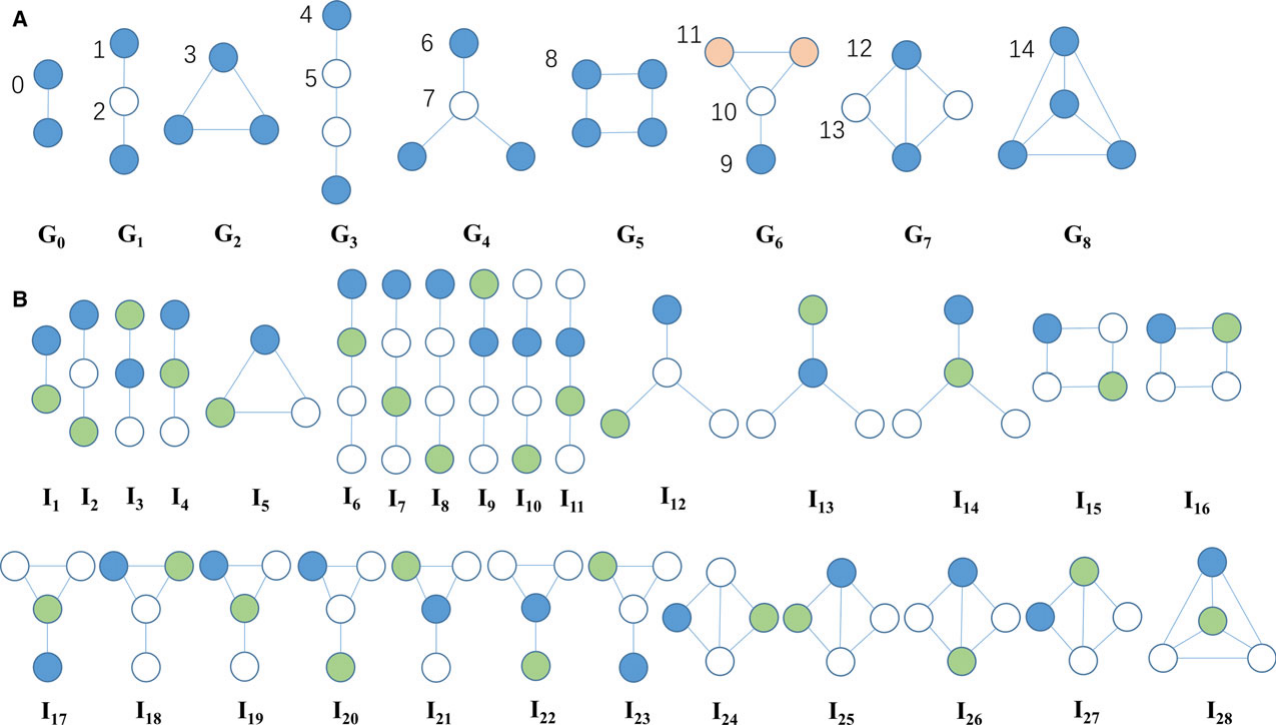
Graphlet types labelled by *G*_0_ to *G*_8_ and automorphism orbits labelled by 0 to 14 (**A**); Graphlet interaction isomers labelled by *I*_1_ to *I*_28_ (**B**). As shown in (**A**), different colours denote different types of orbits in the same graphlet. In (**B**), graphlet interaction is from the blue node to the green one in each isomer.

In this work, based on the disease similarity, the miRNA similarity as well as the known associations between diseases and miRNAs, we proposed a novel method to predict potential miRNA‐disease associations by measuring the graphlet interaction among miRNAs and among diseases. Graphlet is a type of subgraph with a few connections in a large network. In our work, we only considered graphlets with not more than four nodes, which were divided into nine types labelled with *G*_0_ to *G*_8_ in Figure 1A. The position of a node in the graphlet is named automorphism orbit 55. There are totally 15 automorphism orbits in the nine types of graphlets. The relationship between any two nodes in a graphlet is defined as graphlet interaction, which has different types called graphlet interaction isomer according to the different automorphism orbits of the nodes. For the 15 automorphism orbits within nine graphlets, 28 graphlet interaction isomers have been constructed according to 56, which are shown in Figure 1B by labels *I*_1_ to *I*_28_. In particular, if two nodes exchange their positions in a graphlet, the graphlet interaction between them should be regarded as different isomers. In Figure 1B, all graphlet interaction isomers are set from the blue nodes to the green ones. Finally, a graphlet interaction can be described as a vector with 28 elements corresponding to the numbers of 28 isomers 56.

**Figure 2:**
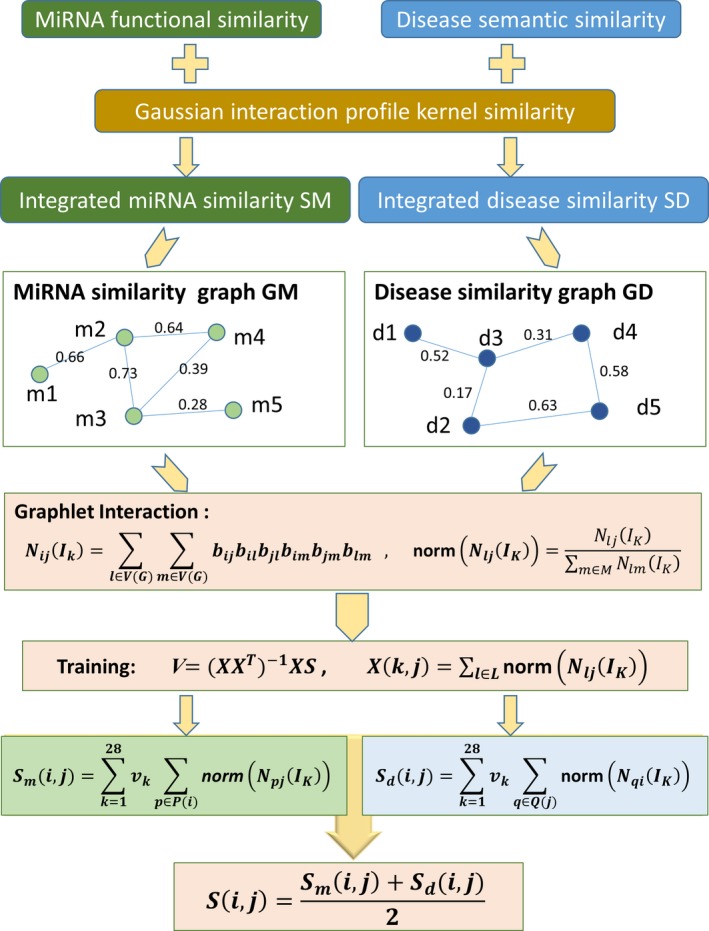
Flow chart of GIMDA model to predict the potential miRNA‐disease associations.

To utilize the disease similarity and miRNA similarity for the prediction of potential associations between diseases and miRNAs, we created a graph *GD* to represent diseases and a graph *GM* to represent miRNAs (Fig. 2). Each node of the graph *GD* denotes a disease and each node of *GM* denotes a miRNA. If there is a similarity between two diseases or two miRNAs, there is an edge between the two corresponding nodes. Furthermore, each edge is assigned a weight with the similarity value between diseases or miRNAs.

In the graph of miRNAs *GM*, we calculated the number of graphlet interaction isomer *I*_*k*_ from node *i* to node *j* as follows 56:

(13) $$N_{\mathrm{ij}}\left(I_{k}\right)=\sum_{l\in V\left(\mathrm{GM}\right)}\sum_{m\in V\left(\mathrm{GM}\right)}b_{\mathrm{ij}}b_{\mathrm{il}}b_{\mathrm{jl}}b_{\mathrm{im}}b_{\mathrm{jm}}b_{\mathrm{lm}}$$

where *V*(*GM*) is the node set of graph *GM*,* l* and *m* denote the other two nodes except node *i* and *j*, and *b* is a variable defined by:

(14) $$b_{\mathrm{st}}=\left\{\begin{array}{cc} a_{\mathrm{st}} & sandthasalinkinI_{k}\\ 1-a_{\mathrm{st}} & sandthasnolinkinI_{k} \end{array}\right.$$

where *a*_*st*_ is the weight of the edge between node *s* and *t*. Particularly, *a*_*st*_ equals to 0 if there is no edge between node *s* and *t*. In the graph of diseases *GD*, the number of graphlet interaction isomer between two diseases can be counted in the same way as described above.

Then, based on the graphlet interaction, we can compute the association score of a miRNA‐disease pair. The computation was implemented in miRNA graph *GM* and in disease graph *GD*, respectively. For the nodes in *GM*, the score of a disease‐miRNA pair (*d*(*i*), *m*(*j*)) can be calculated by the equation:

(15) $$S_{m}\left(i,j\right)=\sum_{k=1}^{28}v_{k}\sum_{p\in P\left(i\right)}\text{norm}\left(N_{\mathrm{pj}}\left(I_{k}\right)\right)$$

where *v*_*k*_ means the weight of the *k*th isomer, *P*(*i*) is the set of miRNAs which have been confirmed to be related to disease *d*(*i*), and norm (*N*_*pj*_ (*I*_*k*_)) is the normalized graphlet interaction calculated by:

(16) $$\text{norm}\left(N_{\mathrm{pj}}\left(I_{k}\right)\right)=\frac{N_{\mathrm{pj}}\left(I_{k}\right)}{\sum_{m\in M}N_{\mathrm{pm}}\left(I_{k}\right)}$$

where *M* is the set of all other miRNAs except *p*. The Eq. (15) can be rewritten in the form of matrix as follows:

(17) $$S_{m}=X_{m}^{T}V_{m}$$

where the matrix *X*_*m*_ is composed of entities given by:

(18) $$X_{m}\left(k,j\right)=\sum_{p\in P\left(i\right)}\text{norm}\left(N_{\mathrm{pj}}\left(I_{k}\right)\right)$$

The weight coefficients *V*_*m*_ can be obtained using linear regression as mentioned in 56. Using the known miRNA‐disease associations as training data set, we can calculate the *X*_*m*_and *S*_*m*_ in Eq. (17). Therefore, the weigh matrix *V*_*m*_ can be computed as follows:

(19) $$V_{m}={\left(X_{m}{X_{m}}^{T}\right)}^{-1}X_{m}S_{m}$$

Similarly, the association score between disease *d*(*i*) and miRNA *m*(*j*) can be calculated in the disease graph *GD*, with the same form as Eq. (15):

(20) $$S_{d}\left(i,j\right)=\sum_{k=1}^{28}v_{k}\sum_{q\in Q\left(j\right)}\text{norm}\left(N_{\mathrm{qi}}\left(I_{k}\right)\right)$$

where *Q*(*j*) is the set of diseases which have been confirmed to be related to miRNA *m*(*j*). Also, Eq. (20) can be transformed into the matrix form as: $S_{d}=X_{d}^{T}V_{d}$,, where *X*_*d*_ was calculated by:

(21) $$X_{d}\left(k,j\right)=\sum_{q\in Q\left(j\right)}\text{norm}\left(N_{\mathrm{qi}}\left(I_{k}\right)\right)$$

Then, we trained the model using known associations to get the weight matrix *V*_*d*_ as Eq. (19):

(22) $$V_{d}={\left(X_{d}{X_{d}}^{T}\right)}^{-1}X_{d}S_{d}$$

Finally, the association score between disease *d*(*i*) and miRNA *m*(*j*) was computed with the average of the scores from *GM* and *GD* as following:

(23) $$S\left(i,j\right)=\frac{S_{m}\left(i,j\right)+S_{d}\left(i,j\right)}{2}$$

## Results

### Performance evaluation

To evaluate the performance of GIMDA, we implemented LOOCV and fivefold cross‐validation using the association data in the HMDD V2.0. In the case of LOOCV, which has two different forms, global and local one, each known association was in turn considered to be the test sample and the others were treated as the training samples. In the global LOOCV, all the miRNA‐disease pairs that have no known associations were treated to be candidate samples, whereas in local LOOCV, candidates only consist of those miRNAs without any known associations with the disease in test sample. We calculated the scores of the test sample and the candidate samples by the GIMDA method. In the local LOOCV, the scores of the test sample and candidate samples including investigated disease were ranked. Whereas in global LOOCV, the test sample was ranked with all candidate samples. In fivefold cross‐validation, all the known miRNA‐disease associations were randomly divided into five equal parts without any overlap between any two of them. Each part was selected in turn as the test samples and the remaining four as training samples. Similarly, all miRNA‐disease pairs without known associations were considered as the candidate samples. Then, the scores of test samples and the candidate samples were computed. We compared the score of each test sample with the scores of candidate samples in turn. The prediction was considered to be successful only when the rank of test sample exceeded the given threshold value.

**Figure 3:**
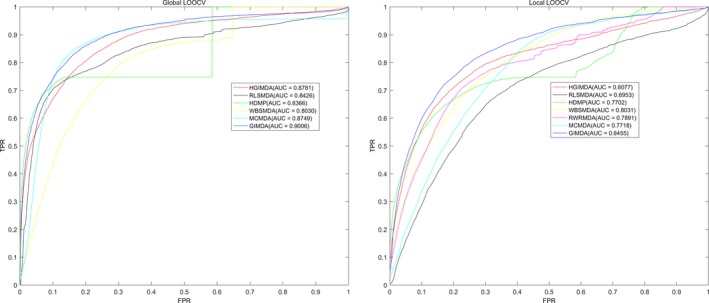
Performance of GIMDA was compared with HGIMDA, RLSMDA, HDMP, WBSMDA and MCMDA in terms of ROC curve and AUC of global leave‐one‐out cross‐validation (LOOCV) (left) and local LOOCV (right). As is shown, GIMDA achieves AUCs of 0.9006 and 0.8455 in the global and local LOOCV, significantly superior to previous models.

Then, correspondingly, we drew the receiver operating characteristics (ROC) curves for three different cross‐validations by plotting true positive rate (TPR, sensitivity) against false‐positive rate (FPR, 1‐specificity) with different thresholds. The sensitivity is the percentage of the test samples whose ranks are above the given threshold, whereas the specificity means the percentage of negative miRNA‐disease associations that are ranked below the given threshold. We calculated the area under the ROC curve (AUC) to evaluate the reliability of the GIMDA. AUC = 1 denotes that the model correctly predicts all test samples, whereas AUC = 0.5 indicates that the model has a random prediction. The AUCs of global and local LOOCV, as well as fivefold cross‐validation of GIMDA are 0.9006, 0.8455 and 0.8927 ± 0.0012, respectively. To compare with previous models, the ROCs and AUCs of HGIMDA, RLSMDA, HDMP, WBSMDA, RWRMDA, MCMDA and GIMDA are shown in Figure 3, from which we can see that the AUC values of GIMDA exceed other models both in global and in local LOOCV. Besides, in fivefold cross‐validation for RLSMDA, HDMP, WBSMDA and MCMDA, the average AUCs and corresponding standard deviations are, in turn, 0.8569 ± 0.0020, 0.8342 ± 0.0010, 0.8185 ± 0.0009 and 0.8767 ± 0.0011. From the comparison, we can conclude that, GIMDA has more reliable prediction ability in searching for potential associations between miRNAs and diseases compared with previous methods.

Additionally, we have performed twofold, threefold and fourfold cross‐validations in the similar way as in fivefold, in which about 50%, 67% and 75% of the training samples, in turn, were taken to re‐train the model, and each process was repeated one hundred times, respectively. As a result, the average AUCs and corresponding standard deviations are 0.8790 ± 0.0052, 0.8873 ± 0.0025 and 0.8904 ± 0.0018 for the three kinds of cross‐validations in turn, which can illuminate the robustness of GIMDA.

### Case studies

Moreover, to further verify the prediction accuracy of GIMDA, we performed case studies for three human complex diseases, colon neoplasms, kidney neoplasms and prostate neoplasms. Top 50 miRNAs of each disease ranked according to their predicted scores were investigated using another two databases, dbDEMC 53 and miR2Disease 54.

Colon neoplasms is one kind of common malignant cancer which has third morbidity and third death rate in the United States 57. It is projected that there will be more than 135,000 individuals newly diagnosed with colon and rectum neoplasms and 50,260 deaths resulted from this disease in the United States in 2017 58, 59. The diagnosis of patients at early stages of colon neoplasms is significant to improve the survival rate. Therefore, it is urgent to develop rapid and sensitive diagnostic markers of the disease. MiRNAs have been reported to be associated with colon neoplasms by many experimental researches. Taking miR‐126 as example 60, a ubiquitous loss of miR‐126 expression has been found in colon neoplasms. This miRNA could target phosphatidylinositol 3‐kinase signalling and inhibit the growth of neoplastic cells. Also, miRNA‐143 was found to have an indirect relationship with the ecotropic viral integration site 1 oncoprotein (Evi1), which led to the low expression of miRNA‐143 in human colon neoplasms 61. We have used GIMDA to predict the top 50 latent associated miRNAs of colon neoplasms and validated them with dbDEMC and miR2Disease. There are 10, 18 and 45 of top 10, top 20 and top 50 predicted miRNAs confirmed by the databases (See Table 1).

**Table 1:** Top 50 miRNAs associated with colon neoplasms were predicted by GIMDA based on HMDD V2.0

miRNA	Evidence	miRNA	Evidence
hsa‐mir‐21	dbdemc; miR2Disease	hsa‐let‐7b	dbdemc; miR2Disease
hsa‐mir‐155	dbdemc; miR2Disease	hsa‐mir‐222	dbdemc
hsa‐mir‐20a	dbdemc; miR2Disease	hsa‐mir‐199a	unconfirmed
hsa‐mir‐146a	dbdemc	hsa‐mir‐133b	dbdemc; miR2Disease
hsa‐mir‐125b	dbdemc	hsa‐mir‐200c	dbdemc; miR2Disease
hsa‐mir‐34a	dbdemc; miR2Disease	hsa‐mir‐15a	dbdemc
hsa‐mir‐16	dbdemc	hsa‐mir‐150	unconfirmed
hsa‐mir‐19b	dbdemc; miR2Disease	hsa‐mir‐10b	dbdemc; miR2Disease
hsa‐mir‐29a	dbdemc; miR2Disease	hsa‐mir‐141	dbdemc; miR2Disease
hsa‐mir‐18a	dbdemc; miR2Disease	hsa‐mir‐181a	dbdemc; miR2Disease
hsa‐mir‐92a	unconfirmed	hsa‐mir‐210	dbdemc
hsa‐mir‐221	dbdemc; miR2Disease	hsa‐mir‐106b	dbdemc; miR2Disease
hsa‐mir‐143	dbdemc; miR2Disease	hsa‐mir‐122	unconfirmed
hsa‐mir‐19a	dbdemc; miR2Disease	hsa‐mir‐107	dbdemc; miR2Disease
hsa‐mir‐1	dbdemc; miR2Disease	hsa‐mir‐182	dbdemc; miR2Disease
hsa‐mir‐200b	dbdemc	hsa‐mir‐22	dbdemc
hsa‐mir‐142	unconfirmed	hsa‐mir‐137	dbdemc; miR2Disease
hsa‐let‐7a	dbdemc; miR2Disease	hsa‐mir‐181b	dbdemc; miR2Disease
hsa‐mir‐29b	dbdemc; miR2Disease	hsa‐mir‐195	dbdemc; miR2Disease
hsa‐mir‐223	dbdemc; miR2Disease	hsa‐mir‐218	dbdemc
hsa‐mir‐30a	miR2Disease	hsa‐mir‐133a	miR2Disease
hsa‐mir‐31	dbdemc; miR2Disease	hsa‐mir‐181b	miR2Disease
hsa‐mir‐9	dbdemc; miR2Disease	hsa‐mir‐24	dbdemc; miR2Disease
hsa‐let‐7e	dbdemc	hsa‐mir‐146b	dbdemc; miR2Disease
hsa‐let‐7c	dbdemc	hsa‐mir‐140	dbdemc; miR2Disease

The top 1‐25 miRNAs are shown in the first column, whereas the top 26‐50 in the second. As a result, 10, 18 and 45 of top 10, top 20 and top 50 were confirmed by the databases, respectively.

Kidney neoplasms, one of the top‐ten cancer killers, is a complex human disease that is hard to detect and treat 62. In 2017, there will be more than 63,000 patients diagnosed with kidney neoplasms and the number of new deaths from this disease will reach to about 14,000 in the United States 59. Kidney neoplasms consists of many types developing from different cell types of the kidney 63, such as chromophobe RCC (chRCC), collecting duct carcinoma (CDC), clear cell RCC (ccRCC) and papillary RCC (PRCC) 64, 65. In the past decades, both our understanding of the genetic basis of the kidney neoplasms and the treatment of patients were improved remarkably 66. However, it is still need to investigate the connections between kidney neoplasms and genetic changes 67. Recent years, many researchers have studied the miRNA expressions of patients suffering from kidney neoplasms in experimental ways. For example, miR‐21 was found up‐regulated in kidney neoplasms 68, whereas five miRNAs including miR‐192, miR‐215, miR‐194, miR‐141 and miR200c, which have a common target gene (ACVR2B), had a lower expression in kidney neoplasms. The GIMDA was applied to predicting associated miRNAs of kidney neoplasms, and the results were validated by dbDEMC and miR2Disease. Finally, 8 of top 10, 16 of top 20 and 45 of top 50 predicted miRNAs were confirmed (See Table 2).

**Table 2:** Top 50 miRNAs associated with kidney neoplasms were predicted by GIMDA based on HMDD V2.0

miRNA	Evidence	miRNA	Evidence
hsa‐mir‐155	Dbdemc	hsa‐mir‐106b	dbdemc; miR2Disease
hsa‐mir‐146a	Dbdemc	hsa‐mir‐101	dbdemc; miR2Disease
hsa‐mir‐34a	dbdemc	hsa‐mir‐34b	dbdemc
hsa‐mir‐92a	unconfirmed	hsa‐mir‐146b	dbdemc
hsa‐mir‐17	miR2Disease	hsa‐mir‐224	dbdemc
hsa‐mir‐16	dbdemc	hsa‐mir‐133b	unconfirmed
hsa‐mir‐29b	dbdemc; miR2Disease	hsa‐mir‐143	dbdemc
hsa‐mir‐221	unconfirmed	hsa‐mir‐182	dbdemc; miR2Disease
hsa‐mir‐20a	dbdemc; miR2Disease	hsa‐mir‐1	dbdemc
hsa‐mir‐18a	dbdemc	hsa‐mir‐122	dbdemc; miR2Disease
hsa‐mir‐29a	dbdemc; miR2Disease	hsa‐mir‐34c	dbdemc
hsa‐mir‐145	dbdemc	hsa‐mir‐27a	dbdemc; miR2Disease
hsa‐mir‐19b	dbdemc; miR2Disease	hsa‐mir‐218	dbdemc
hsa‐mir‐222	dbdemc	hsa‐mir‐127	dbdemc
hsa‐mir‐19a	dbdemc	hsa‐mir‐15b	dbdemc
hsa‐mir‐125b	unconfirmed	hsa‐mir‐24	dbdemc
hsa‐mir‐133a	unconfirmed	hsa‐mir‐183	dbdemc
hsa‐mir‐195	dbdemc	hsa‐mir‐223	dbdemc
hsa‐mir‐210	dbdemc; miR2Disease	hsa‐mir‐181a	dbdemc
hsa‐mir‐199a	dbdemc; miR2Disease	hsa‐mir‐26a	dbdemc; miR2Disease
hsa‐mir‐126	dbdemc; miR2Disease	hsa‐let‐7a	dbdemc
hsa‐mir‐181b	dbdemc	hsa‐mir‐196a	dbdemc
hsa‐mir‐93	dbdemc	hsa‐mir‐200a	dbdemc
hsa‐mir‐31	dbdemc	hsa‐mir‐9	dbdemc
hsa‐mir‐200b	dbdemc; miR2Disease	hsa‐mir‐29c	dbdemc; miR2Disease

The top 1‐25 miRNAs are shown in the first column, whereas the top 26‐50 in the second. As a result, 8, 16 and 45 of top 10, top 20 and top 50 were confirmed by the databases, respectively.

Prostate neoplasms is one of the biggest threats to men's health in the worldwide. More than 26,000 deaths are caused by prostate neoplasms every year in the United States 57, 59. Its incidence is strongly related to age and has a higher rate in older man. But recently, more and more diagnoses occur in younger man 69. Prostate neoplasms may spread to other parts of human body, preferentially to regional lymph nodes and bones. It has become relatively easier to detect and stage prostate neoplasms, monitor response of patients to treatment and detect recurrence since using serum prostate specific antigen (PSA) screening 70. However, elevated PSA levels may be confounded by other factors; therefore, it is still necessary to develop sensitive and specific biomarkers of prostate neoplasms for early diagnosis. MiRNAs proposed as a biomarker of prostate neoplasms have attracted more and more attentions in the past few years 71, 72. For instance, miR‐145 was found consistently down‐regulated in prostate neoplasms 73. MiR‐145 targets 3′ untranslated region (UTR) of ERG mRNA and its down‐regulation may contribute to the increased expression of most ERG splice variants sharing the miR‐145 target sequence in their 3′‐UTR. Moreover, it was found that the expression of miR‐574‐3p was significantly lower in prostate neoplasms 74. GIMDA was also used for predicting potential associated miRNAs of prostate neoplasms. For the top 10, top 20 and top 50 predicted miRNAs, there are 9, 18 and 41 miRNAs verified by databases, respectively (See Table 3).

**Table 3:** Top 50 miRNAs associated with prostate neoplasms were predicted by GIMDA based on HMDD V2.0

miRNA	Evidence	miRNA	Evidence
hsa‐mir‐21	dbdemc; miR2Disease	hsa‐mir‐19a	dbdemc
hsa‐mir‐146a	miR2Disease	hsa‐mir‐195	dbdemc; miR2Disease
hsa‐mir‐155	Dbdemc	hsa‐mir‐200b	unconfirmed
hsa‐mir‐34a	dbdemc; miR2Disease	hsa‐mir‐1	dbdemc
hsa‐mir‐17	miR2Disease	hsa‐mir‐15b	dbdemc
hsa‐mir‐20a	miR2Disease	hsa‐mir‐101	dbdemc; miR2Disease
hsa‐mir‐92a	Unconfirmed	hsa‐mir‐224	dbdemc; miR2Disease
hsa‐mir‐29a	dbdemc; miR2Disease	hsa‐mir‐214	dbdemc; miR2Disease
hsa‐mir‐16	dbdemc; miR2Disease	hsa‐mir‐34c	dbdemc
hsa‐mir‐29b	dbdemc; miR2Disease	hsa‐mir‐335	unconfirmed
hsa‐mir‐221	dbdemc; miR2Disease	hsa‐mir‐133b	dbdemc
hsa‐mir‐222	dbdemc; miR2Disease	hsa‐mir‐182	dbdemc; miR2Disease
hsa‐mir‐15a	dbdemc; miR2Disease	hsa‐mir‐203	unconfirmed
hsa‐mir‐126	dbdemc; miR2Disease	hsa‐mir‐93	unconfirmed
hsa‐mir‐18a	Unconfirmed	hsa‐mir‐124	dbdemc
hsa‐mir‐223	dbdemc; miR2Disease	hsa‐mir‐106a	dbdemc; miR2Disease
hsa‐mir‐19b	dbdemc; miR2Disease	hsa‐mir‐148a	miR2Disease
hsa‐mir‐31	dbdemc; miR2Disease	hsa‐mir‐486	unconfirmed
hsa‐mir‐199a	dbdemc; miR2Disease	hsa‐mir‐210	miR2Disease
hsa‐mir‐133a	Dbdemc	hsa‐mir‐26a	dbdemc; miR2Disease
hsa‐mir‐143	dbdemc; miR2Disease	hsa‐let‐7a	dbdemc; miR2Disease
hsa‐mir‐122	Unconfirmed	hsa‐mir‐34b	dbdemc
hsa‐mir‐181b	dbdemc; miR2Disease	hsa‐mir‐200a	dbdemc
hsa‐mir‐106b	Dbdemc	hsa‐mir‐218	dbdemc; miR2Disease
hsa‐mir‐146b	Unconfirmed	hsa‐mir‐127	dbdemc; miR2Disease

The top 1‐25 miRNAs are shown in the first column, whereas the top 26‐50 in the second. As a result, 9, 18 and 41 of top 10, top 20 and top 50 were confirmed by the databases, respectively.

The whole prediction list of potential miRNAs associated with each disease in the database of HMDD V2.0 was provided in Table S1, which was ranked according to the association scores calculated by GIMDA. We hope that our prediction results can provide guidance for biological experiments and can be validated by more experimental studies.

Besides, to manifest the predictive ability of GIMDA for new diseases without any known related miRNAs, we removed all the existing associations between the investigated disease and miRNAs. Then, we computed the association scores of all miRNAs for this disease. The results of hepatocellular carcinoma confirmed by HMDD V2.0, dbDEMC and miR2Disease are shown in Table 4, from which we can see that 10, 20 and 50 of top 10, top 20 and top 50 predicted miRNAs were confirmed by at least one of the three databases. For example, hsa‐mir‐21 which ranks first in the list has been reported to have relation with hepatoma cell growth by experiment 75.

**Table 4:** Top 50 miRNAs associated with carcinoma, hepatocellular were predicted by GIMDA with hiding all known related miRNAs based on HMDD V2.0

miRNA	Evidence	miRNA	Evidence
hsa‐mir‐21	miR2Disease;HMDD	hsa‐mir‐1	miR2Disease;HMDD
hsa‐mir‐155	dbdemc;miR2Disease;HMDD	hsa‐mir‐29a	dbdemc;HMDD
hsa‐mir‐17	miR2Disease;HMDD	hsa‐mir‐181b	dbdemc;miR2Disease;HMDD
hsa‐mir‐20a	dbdemc;miR2Disease;HMDD	hsa‐mir‐142	miR2Disease;HMDD
hsa‐mir‐34a	dbdemc;miR2Disease;HMDD	hsa‐mir‐9	miR2Disease
hsa‐mir‐125b	miR2Disease;HMDD	hsa‐mir‐200b	miR2Disease;HMDD
hsa‐mir‐221	dbdemc;miR2Disease;HMDD	hsa‐mir‐29b	dbdemc;HMDD
hsa‐mir‐19b	miR2Disease;HMDD	hsa‐mir‐200a	dbdemc;miR2Disease;HMDD
hsa‐mir‐92a	miR2Disease;HMDD	hsa‐mir‐150	dbdemc;miR2Disease;HMDD
hsa‐mir‐18a	dbdemc;miR2Disease;HMDD	hsa‐let‐7b	miR2Disease;HMDD
hsa‐mir‐16	dbdemc;miR2Disease;HMDD	hsa‐mir‐31	miR2Disease;HMDD
hsa‐mir‐145	dbdemc;miR2Disease;HMDD	hsa‐let‐7e	dbdemc;miR2Disease;HMDD
hsa‐mir‐146a	dbdemc;miR2Disease;HMDD	hsa‐mir‐205	miR2Disease;HMDD
hsa‐mir‐126	dbdemc;miR2Disease;HMDD	hsa‐mir‐182	miR2Disease;HMDD
hsa‐mir‐223	miR2Disease;HMDD	hsa‐let‐7i	dbdemc;HMDD
hsa‐mir‐19a	dbdemc;miR2Disease;HMDD	hsa‐mir‐10b	HMDD
hsa‐mir‐181a	dbdemc;miR2Disease;HMDD	hsa‐mir‐106b	dbdemc;miR2Disease;HMDD
hsa‐mir‐222	dbdemc;miR2Disease;HMDD	hsa‐let‐7c	dbdemc;miR2Disease;HMDD
hsa‐mir‐143	dbdemc;miR2Disease	hsa‐mir‐24	miR2Disease;HMDD
hsa‐let‐7a	dbdemc;miR2Disease;HMDD	hsa‐mir‐146b	HMDD
hsa‐mir‐203	miR2Disease;HMDD	hsa‐mir‐195	dbdemc;miR2Disease;HMDD
hsa‐mir‐29c	dbdemc;HMDD	hsa‐mir‐100	dbdemc;HMDD
hsa‐mir‐15a	dbdemc;miR2Disease;HMDD	hsa‐mir‐199a	dbdemc;miR2Disease;HMDD
hsa‐mir‐210	dbdemc;HMDD	hsa‐mir‐30a	miR2Disease;HMDD
hsa‐mir‐200c	HMDD	hsa‐mir‐34c	HMDD

The top 1‐25 miRNAs are shown in the first column, whereas the top 26‐50 in the second. As a result, 10, 20 and 50 of top 10, top 20 and top 50 were confirmed by the databases, respectively.

To investigate the robustness of GIMDA prediction performance, we implemented the model on the database HMDD V1.0. The result suggests that the GIMDA is very effective on the prediction of potential associations between miRNAs and diseases even using different data sets. Table 5 shows the top 50 miRNAs related to oesophageal neoplasms, which were predicted by GIMDA based on the HMDD V1.0. As is shown in Table 5, there are 10, 20 and 49 of top 10, top 20 and top 50 miRNAs confirmed by at least one of the three databases mentioned above. For instance, the highest score in the list is obtained by the hsa‐mir‐155. It has been found that the relative expressions of miR‐155 in oesophageal tissue were significantly associated with increased risk for oesophageal neoplasms and the circulating miR‐155 had significant diagnostic value for oesophageal neoplasms 76.

**Table 5:** Top 50 miRNAs associated with oesophageal neoplasms were predicted by GIMDA based on HMDD V1.0

miRNA	Evidence	miRNA	Evidence
hsa‐mir‐17	Dbdemc	hsa‐mir‐199a	dbdemc;HMDD
hsa‐mir‐20a	dbdemc;HMDD	hsa‐mir‐146b	dbdemc
hsa‐mir‐155	dbdemc;HMDD	hsa‐let‐7f	unconfirmed
hsa‐mir‐18a	Dbdemc	hsa‐mir‐181a	dbdemc
hsa‐let‐7a	dbdemc;HMDD	hsa‐mir‐29c	dbdemc;HMDD
hsa‐mir‐19a	dbdemc;HMDD	hsa‐mir‐141	dbdemc;HMDD
hsa‐mir‐16	Dbdemc	hsa‐let‐7 g	dbdemc
hsa‐mir‐221	Dbdemc	hsa‐mir‐127	dbdemc
hsa‐mir‐19b	Dbdemc	hsa‐mir‐29b	dbdemc
hsa‐mir‐15a	dbdemc;HMDD	hsa‐mir‐15b	dbdemc
hsa‐mir‐92a	HMDD	hsa‐mir‐106b	dbdemc
hsa‐mir‐146a	dbdemc;HMDD	hsa‐mir‐106a	dbdemc
hsa‐mir‐223	dbdemc;miR2Disease;HMDD	hsa‐mir‐200a	dbdemc;HMDD
hsa‐mir‐145	dbdemc;HMDD	hsa‐mir‐34a	dbdemc;HMDD
hsa‐mir‐200b	Dbdemc	hsa‐mir‐132	dbdemc
hsa‐mir‐222	Dbdemc	hsa‐mir‐194	dbdemc;miR2Disease
hsa‐let‐7b	dbdemc;HMDD	hsa‐mir‐30c	dbdemc
hsa‐let‐7e	Dbdemc	hsa‐mir‐1	dbdemc
hsa‐let‐7c	dbdemc;HMDD	hsa‐mir‐196a	dbdemc;miR2Disease;HMDD
hsa‐let‐7d	Dbdemc	hsa‐mir‐214	dbdemc;HMDD
hsa‐mir‐125b	Dbdemc	hsa‐mir‐125a	dbdemc
hsa‐mir‐126	dbdemc;HMDD	hsa‐mir‐205	dbdemc;miR2Disease;HMDD
hsa‐mir‐181b	Dbdemc	hsa‐mir‐99b	dbdemc;HMDD
hsa‐let‐7i	Dbdemc	hsa‐mir‐9	dbdemc
hsa‐mir‐143	dbdemc;HMDD	hsa‐mir‐23b	dbdemc

The top 1‐25 miRNAs are shown in the first column, whereas the top 26‐50 in the second. As a result, 10, 20 and 49 of top 10, top 20 and top 50 were confirmed by the databases, respectively.

## Discussion

In this study, we presented a prediction model named GIMDA to discover latent miRNA‐disease associations based on graphlet interaction with integrating disease semantic similarity, miRNA functional similarity, Gaussian interaction profile kernel similarity and known miRNA‐disease associations. In GIMDA, all miRNAs are denoted as nodes of a graph, although all diseases are denoted as nodes of another graph. If two nodes in the same graph have a similarity, there is an edge between them, otherwise not. Each edge was assigned a weight using the similarity value between two miRNAs or between two diseases. The graphlet is a type of small‐connected subgraph that is non‐isomorphic. Graphlet interaction describes the relationship between two nodes in the graph 56. By calculating the number of each graphlet interaction isomer from the node that has known association to the node without known association, we can compute the association score of a miRNA‐disease pair. The prediction performance of GIMDA was represented by implementing global and local LOOCV, as well as fivefold cross‐validation. Additionally, three different types of case studies were applied to several complex human diseases. The results from both cross‐validations and case studies have shown that GIMDA performed outstandingly in predicting potential miRNA‐disease associations.

There are some reasons that account for the reliable performance of this model. First of all, GIMDA predicted potential associations between miRNAs and diseases based on the abundant data obtained from HMDD V2.0. Besides, the model integrated Gaussian interaction profile kernel similarity with miRNA functional similarity and disease semantic similarity, which made the similarity between two miRNAs or two diseases more precision. Moreover, GIMDA described the complex relationship between two nodes based on graphlet interaction, in which both direct and indirect links between the nodes were considered. Finally, this method combined the association score of a miRNA‐disease pair calculated in the miRNA graph with the score calculated in the disease graph, which made it applicable to predict new diseases without any known related miRNAs or new miRNAs without any known related diseases.

Whereas there are some deficiencies of GIMDA. Firstly, the number of known miRNA‐disease associations is still not sufficient. In the future, when the data set is expanded, the model will perform better. Secondly, GIMDA calculated the graphlet interaction only considering the graphlets that contained no more than four nodes, which means that the calculation excluded the similarity information of those nodes that were linked to each other indirectly more than three edges. We will take more nodes into account when the computing conditions are improved in the future. Thirdly, GIMDA is helpless for predicting the total new association between a new miRNA and a new disease. In addition, in this model, we counted the graphlet interaction between any two nodes in the same graph for 28 types of isomers in the miRNA graph and the diseases graph, respectively, which was somewhat time‐consuming in current situation. Furthermore, as is known, taking use of graphlet or network motif has obtained excellent results in modelling and prediction for many biological problems 55, 56, 77, 78, 79, and the subnetwork can locally reveal the dynamic properties and improve our understanding of the function of the whole network 78. In the context, we think that it is feasible and necessary to develop effective computational methods based on the concept of graphlet or network motif for miRNA‐disease association prediction in the future. Finally, we are expecting that method that is more rational will be developed to evaluate the performance of the models for miRNA‐disease association prediction, such as the re‐sampling test which can estimate the robustness of the models 80.

## Conflicts of interest

The authors declare no conflict(s) of interest.

## Supporting information


**Table S1.** The whole prediction list of all candidate miRNA‐disease pairs ranked according to the calculated association scores.
