# Supplementary material for: GIMDA: Graphlet interaction‐based MiRNA‐disease association prediction
Source: J Cell Mol Med. 2017 Dec 22;22(3):1548–61. doi: 10.1111/jcmm.13429 (PMC5824414; doi:10.1111/jcmm.13429)
Supplement: Supplementary file 2 [file JCMM-22-1548-s002.docx]

**GIMDA: Graphlet Interaction-based MiRNA-Disease Association prediction**

Xing Chen^1,^*, Na-Na Guan^2^, Jian-Qiang Li^2,^* , Gui-Ying Yan^3^

^1^School of Information and Control Engineering, China University of Mining and Technology, Xuzhou, 221116, China

^2^College of Computer Science and Software Engineering, Shenzhen University, Shenzhen, 518060, China

^3^Academy of Mathematics and Systems Science, Chinese Academy of Sciences, Beijing, 100190, China

*Corresponding author

**Email**: [xingchen@amss.ac.cn](mailto:xingchen@amss.ac.cn); [lijq@szu.edu.cn](mailto:lijq@szu.edu.cn)

**Supplementary Information**

**Supplementary Table 1.** The whole prediction list of all candidate miRNA-disease pairs ranked according to the calculated association scores. The prediction scores are calculated by GIMDA based on all the known miRNA-disease associations in HMDD database. This prediction result is released for further experimental validation and research.
